# The utilization of activated carbon derived from polyethylene terephthalate bottle waste, as a sustainable source for removal and recovery of methylene blue and picric acid from aqueous solutions

**DOI:** 10.1186/s13065-026-01728-x

**Published:** 2026-02-28

**Authors:** Asmaa Halima, Magdi E. Khalifa, Nasser Mohammed Hosny, Wael I. Mortada

**Affiliations:** 1 Department of Chemistry, Faculty of Science, Mansoura University, Mansoura, 35516 Egypt; 2 Chemistry Department, Faculty of Science, Port Said University, P.O. Box 4252, Port-Said, Egypt; 3 Urology and Nephrology Center, Mansoura University, Mansoura, 35516 Egypt

**Keywords:** Waste PET bottles, Activated carbon, KOH activation, Solid phase extraction, Methylene blue, Picric acid

## Abstract

In this study, waste polyethylene terephthalate (PET) from discarded bottles was recycled via KOH treatment to produce activated carbon (AC) to remove methylene blue (MB) and picric acid (PA) from contaminated water. The AC produced was characterized by using physicochemical methods to assess its surface porosity, morphology and function groups. Batch adsorption experiments were performed the impact of pH, sorbent amount, time, sample volume, and initial concentration of the target dye on the adsorption efficiency. Adsorption followed the Langmuir isotherm model indicating monolayer adsorption behavior, while kinetics fitted with the pseudo-second-order model. The prepared AC exhibited high maximum adsorption capacities reached 334.4 mg g⁻¹ for MB and 271.7 mg g⁻¹ for PA. Quantitative dye desorption (> 97% recovery) was achieved using H₂SO₄ for MB and NaOH for PA. The method was also applied to spectrophotometric quantification of both analytes, yielding linear calibration curves for MB (0.15–5.76 mg L⁻¹, R² = 0.9997) and PA (0.01–1.68 mg L⁻¹, R² = 0.9980). Detection limits were 0.04 mg L⁻¹ (MB) and 0.003 mg L⁻¹ (PA), with quantification limits of 0.15 mg L⁻¹ (MB) and 0.013 mg L⁻¹ (PA). Successful application to diverse water samples confirmed the method’s efficacy for dye removal and quantification.

**Supplementary Information:**

The online version contains supplementary material available at 10.1186/s13065-026-01728-x.

## Introduction

 Dye pollution poses a significant environmental challenge, with over 100,000 commercially available dyes and annual production exceeding one million tons, more than 20% of which are discharged untreated into the environment, leading to severe ecological disruption and marine pollution [1]. Among this vast array of pollutants, Methylene Blue (MB) and Picric Acid (PA) have been selected for this study as representative models of cationic and anionic dyes, respectively, owing to their high toxicity and documented environmental impact. MB, a widely used heterocyclic compound, is applied in medicine, paper pigmentation, coatings, and the dyeing of tissue paper substrates, among other uses. MB poses significant risks to human health, causing both localized irritation (gastrointestinal, dermal, ocular) and systemic effects such as nausea, vomiting, tachycardia, dyspnea, and cyanosis. Furthermore, its neurotoxic potential is evidenced by symptoms like dizziness, confusion, and headaches in adults, a finding consistent with demonstrated neurotoxicity in animal models [2]. Numerous industries, including those in leather, plastics, fireworks, explosives, dyes, and pharmaceuticals, use PA. Due to its sublimation at room temperature and volatility, PA poses serious risks to human health and the environment. Exposure to PA may cause respiratory issues, skin and eye irritation, and, at high doses, more severe conditions such as kidney failure, liver damage, abdominal pain, and potentially cancer [3, 4]. Exposure to MB and PA occurs through contaminated water, skin contact, inhalation, ingestion, and uptake by aquatic organisms and soil systems, mainly due to industrial wastewater discharge [5].

Treating contaminated effluents containing dyes is critical to prevent environmental pollution. Various methods have been developed to remove organic pollutants from water, such as membrane separation [6], coagulation [7], flocculation [8], biodegradation [9], ion exchange [10], ozonation [11] and oxidation [12]. However, these conventional techniques often suffer from several limitations, including high operational costs, membrane fouling, generation of secondary sludge, and incomplete removal of pollutants at low concentrations. In contrast, adsorption (solid-phase extraction, SPE) has gained significant attention due to its simplicity, low cost, high efficiency, and environmental compatibility [13]. This process removes dye molecules without generating toxic byproducts or residues, making it a promising and sustainable approach for wastewater treatment [14]. SPE is an essential technique in analytical chemistry used for the concentration and purification of trace analytes [15]. It is a sorbent-based method that selectively adsorbs analytes onto a solid phase, which are then eluted with a suitable solvent. This technique offers high selectivity and reproducibility while minimizing solvent usage [16].

Activated carbon (AC) is vital in industrial processes such as liquid and gas separation, solvent recovery, toxic substance removal, water purification, and use as catalysts or catalyst supports [17]. AC with high specific surface areas can be produced from various carbonaceous sources, including coal [18], nutshells [19], wood [20], agricultural residues [21], industrial wastes [22], plant biomass [23], and goat-dropping [24]. Among domestic wastes, polyethylene terephthalate (PET) stands out due to its rapidly growing consumption in the global plastic market, driven largely by the widespread use of PET bottles [25]. The poor biodegradability and photodegradability of PET pose significant environmental challenges, making its conversion into AC an environmentally sustainable recycling solution [26]. The utilization of PET as a precursor for AC produces numerous significant advantages. PET is a prevalent and inexpensive plastic waste, rendering it a cost-efficient and sustainable carbon source. The elevated carbon content and appropriate chemical structure facilitate the development of extensive porous networks during activation, resulting in activated carbon with a substantial surface area and robust adsorption capability. Moreover, transforming PET trash into activated carbon mitigates plastic pollution while generating a valuable adsorbent substance [27].

There are two primary methods for preparing AC: chemical activation and physical activation. Chemical activation offers benefits such as improved pore structure, increased yield, and lower activation temperatures. This process involves impregnating raw materials with chemicals such as phosphoric acid and potassium hydroxide to create mesopores and micropores. Furthermore, it requires less energy and time, making chemically activated carbon highly customizable for various applications [28].

This study developed an efficient solid-phase extraction (SPE) procedure using a cost-effective activated carbon (AC) derived from recycled PET waste. The prepared AC was thoroughly characterized using FT-IR, XPS, SEM/EDX, BET surface area analysis, and XRD. The adsorption behavior of methylene blue (MB) and picric acid (PA) was investigated, and the mechanism was elucidated through pHPZC, FT-IR, and thermodynamic studies. The innovative aspect of this material lies in its ability to not only remove MB and PA but also to enable their efficient recovery. This study enhances adsorbent reusability, reduces secondary waste, and improves detection sensitivity via preconcentration. The developed procedure was successfully employed for the preconcentration and spectrophotometric quantification of the target analytes, demonstrating good analytical performance and highlighting the material’s significant potential for applications in wastewater treatment and environmental monitoring.

## Materials and methods

### Chemicals

Analytical grade chemicals were used through this work. Methylene blue (MB, ~ 99%), potassium hydroxide (KOH pellets, ~ 85%), picric acid (PA, ~ 99.5%), sodium hydroxide (NaOH pellets, ~ 97%), sulfuric acid (H₂SO₄, 98%), and hydrochloric acid (HCl, 37%) were supplied by Alpha Chemicals Private Limited (Mumbai, India) and used without further purification. The other chemicals were obtained from Sigma-Aldrich (Shanghai, China). All buffer solutions were prepared according to standard methods [29, 30]. Specifically, glycine buffer (pH 1.0) using glycine and HCl, citrate buffer (pH 2.0–3.0) from citric acid and sodium citrate, acetate buffer (pH 4.0–6.0) prepared with acetic acid and sodium acetate, and phosphate buffer (pH 7.0–8.0) using sodium dihydrogen phosphate (NaH₂PO₄) and disodium hydrogen phosphate (Na₂HPO₄).

### Synthesis of activated carbon

Waste PET bottles were collected, thoroughly washed with deionized water, dried at 70 °C, and cut into 1–3 mm fragments. Then, 10 g of PET was mixed with KOH at a mass impregnation ratio of 1:1 and agitated at 85–95 °C for 6 h to facilitate chemical diffusion. The impregnated PET was subsequently heated in a quartz tube furnace under a nitrogen gas stream (50 mL/min) at 400 °C for 1 h, followed by a second heating stage at 800 °C for an additional hour (using a VULCAN 3–550, 120 V furnace). After cooling, the residue was treated with 250 mL of 1.0 M HCl for 1 h and thoroughly rinsed with hot distilled water until the washing solution reached a neutral pH. The final product was dried in an electrical oven at 110 °C overnight, to ensure the removal of moisture or gas contamination. Previous studies showed that adjusting the KOH-to-PET mass ratio plays a key role in the process, with a 1:1 ratio delivering the best results. This balance not only boosts activation efficiency but also dramatically increases surface area, which are crucial for maximizing the carbon material’s adsorption performance [28].

### Characterization

The physicochemical properties of the synthesized carbon material were characterized using several analytical techniques. X-ray diffraction (XRD) analysis was performed using a D8 Advance diffractometer (Bruker AXS, Germany) with Cu Kα radiation (λ = 0.1540 nm), scanning a 2θ range from 3° to 80° with a step size of 0.02°. Surface elemental composition and chemical states were analyzed by X-ray photoelectron spectroscopy (XPS) using a K-Alpha spectrometer (Thermo Scientific, USA) equipped with a monochromatic Al Kα source. Functional groups were identified by Fourier Transform Infrared (FT-IR) spectroscopy using an iS10 spectrometer (Thermo Nicolet, USA) with KBr pellets, covering a wavenumber range of 4000 to 400 cm⁻¹. Finally, the material’s morphology and elemental composition were examined by scanning electron microscopy with energy-dispersive X-ray spectroscopy (SEM/EDX) using a Quanta 250 FEG microscope (FEI, USA). Surface area and pore size distribution were analyzed using a BELSORP-mini X analyzer (Microtrac BEL Corp., Japan) via Brunauer-Emmett-Teller (BET) method. Prior to analysis, samples were degassed under nitrogen flow at 200 °C for 12 h. The point of zero charge (pH_PZC_) was determined using the pH drift method [31]. Briefly, 20.0 mg of AC was added to a series of bottles containing 50 mL of 0.1 mol L⁻¹ NaCl solution. The initial pH (pH_initial_) was adjusted to values between 2.0 and 10.0 using 0.1 mol L⁻¹ HCl or NaOH solutions. The suspensions were agitated at 150 rpm and maintained at 25.0 ± 1.0 °C for 24 h and the pH was measured again (pH_final_). The pH difference (ΔpH = pH_final_- pH_initial_) was plotted against pH_initial_, with the pHPZC identified as the intersection point of the curve with the x-axis.

### The adsorption experiments

Batch experiments were conducted to optimize the adsorption process by evaluating the effects of temperature (298–328 K), contact time (5–240 min), sorbent dose (0.001–0.02 g), solution pH (1.0–8.0, adjusted with buffer solutions), and solution volume (10–250 mL). In a typical procedure, a certain amount of the adsorbent is mixed with a solution containing the analyte under controlled conditions. The mixture is stirred for a specific time. After phase separation by centrifugation (3000 rpm, 10 min), the remaining concentration of the analyte in the solution is measured spectrophotometrically at their respective maximum absorption wavelengths (662 nm for MB and 348 nm for PA) using a Genway 7300 UV-Vis spectrophotometer (Cole-Parmer Ltd., Staffordshire, UK). The adsorption efficiency was assessed by calculating both the removal percentage (R%) and equilibrium adsorption capacity (qₑ, mg g⁻¹) using Eqs. 1 and 2.

1 $$\mathrm{R}\:\left(\mathrm{\%}\right)=\frac{{C}_{i}-{C}_{e}}{{C}_{i}}\:\times\:100$$

2 $$\:\:\:\:\:\:{q}_{e}=\frac{\left(Ci-\:Ce\right)V}{m}$$

The initial and equilibrium concentrations of MB and PA solutions (expressed in mg L⁻¹) are denoted as C_i_ and Cₑ, respectively. The total volume of the aqueous solution is represented by V (in liters), and the sorbent mass is designated as m (in grams).

### The study of desorption

A desorption experiment was conducted to evaluate the reusability of the adsorbent. For this purpose, 10 mg of AC was soaked in 20 mL of a 100 mg L⁻¹ solution of either MB or PA. The mixture was shaken for 180 min (MB) or 90 min (PA) to reach adsorption equilibrium. The adsorbent was then separated, washed with distilled water to remove any unbound dye, and treated with 2.0 mL of eluent (0.5 mol L⁻¹ H₂SO₄ for MB or 0.5 mol L⁻¹ NaOH for PA). The mixture was stirred for 30 min, rinsed with distilled water, dried, and subjected to another adsorption-desorption cycle to assess the regeneration efficiency and reusability of the AC. The dye concentration in the filtrate was measured using a UV-Vis spectrophotometer, as described previously.

### Selectivity study

The method’s selectivity was evaluated by adding various concentrations of common ions and interfering dyes to 20 mL of a 1.0 mg L⁻¹ solution of either MB or PA. Each spiked sample was processed using the optimized extraction procedure. The concentration of each interfering species increased until the recovery deviated by more than ±5%. The highest concentration that maintained an acceptable recovery (95.0–105.0.0.0%) was designated as the tolerance limit.

### Adsorption isotherm

The adsorption isotherm demonstrates the relationship between the adsorption capacity and the equilibrium concentration of the adsorbate. To construct the isotherm, 10 mg of AC was mixed with 20 mL of MB or PA solutions at varying concentrations (25–400 mg L⁻¹) and stirred at room temperature for 180 min (MB) or 90 min (PA). After adsorption, the adsorbent was separated, and the residual concentrations of MB and PA (Cₑ) were determined spectrophotometrically. The Langmuir, Freundlich, Temkin, and Dubinin–Radushkevich (D–R) isotherm models were applied to analyze the adsorption data using Eqs. (3), (4), (5), and (6) respectively.

3 $$\:\frac{{C}_{e}}{{q}_{e}}=\:\frac{{C}_{e}}{{q}_{max}}+\frac{1}{{q}_{\mathrm{max}{K}_{L}}}$$

4 $$\text {Ln} \:{\mathrm{q}}_{\mathrm{e}\:}=\mathrm{ln}\mathrm{K}\mathrm{F}+\frac{1}{\mathrm{n}\mathrm{F}}\mathrm{ln}{\mathrm{C}}_{\mathrm{e}}$$

5 $$\:{q}_{e}=\frac{R\:T}{{b}_{t}}\mathrm{ln}{A}_{t\:}+\frac{R\:T}{{b}_{t}}\mathrm{ln}{C}_{e}$$

6 $$\mathrm{ln}{q}_{e}=\mathrm{ln}{q}_{m}-\beta{\epsilon}^{2}$$

here, Cₑ represents the equilibrium concentration (mg L⁻¹) of the adsorbate in the solution, while qₑ denotes the amount of dye adsorbed onto AC at equilibrium (mg g⁻¹). qₘₐₓ is the maximum adsorption capacity of AC for MB and PA (mg g⁻¹), while K_L_, KF, and Aₜ are the Langmuir, Freundlich, and Temkin constants (L mg⁻¹), respectively. The heterogeneity factor in the Freundlich model is indicated by 1/nF. A value between 0 and 1 corresponds to favorable adsorption conditions, where values closer to zero suggest high surface heterogeneity (indicating irregular adsorption sites), while values approaching 1 indicate more homogeneous adsorption sites. qₘ is the theoretical monolayer capacity, β is a constant related to the adsorption energy, and ε = RT ln(1 + 1/Cₑ) is the Polanyi potential.

### Thermodynamic parameters

The Vant Hoff formula was used to calculate changes in entropy (ΔS) and enthalpy (ΔH):

7 $$\mathrm{ln}{\mathrm{K}}_{\mathrm{d}}=\frac{\Delta\mathrm{S}}{\mathrm{R}}-\frac{\Delta\mathrm{H}}{\mathrm{R}\mathrm{T}}$$

Here, K_d_ (the adsorption equilibrium constant, in L g⁻¹) is calculated as K_d_ = qₑ/Cₑ. T represents the absolute temperature (in Kelvin), and R is the universal gas constant (8.314 J·mol⁻¹·K⁻¹). By plotting ln K_d_ versus 1/T, the slope and intercept of the linear fit were used to determine ΔH and ΔS, respectively. The Gibbs free energy change (ΔG) for the adsorption of MB and PA at 298, 308, 318, 328 K was then calculated using the following equation:

8 $$\Delta G = \Delta H - T\Delta S$$

### Real water samples

To evaluate the efficiency of the preconcentration process, the extraction of MB and PA was investigated using real water samples collected from various locations in Mansoura, Egypt. The samples included treated sewage wastewater, Nile River water, and tap water. Prior to analysis, all samples were filtered through 0.45 μm cellulose membranes to remove suspended particulates. The characteristics of the real sample are presented in Table 1S. The developed procedure was then applied to 20 mL aliquots of each water sample to assess method applicability under realistic environmental conditions. Preconcentration efficiency was quantitatively assessed by calculating the recovery percentage (%R) using Eq. (1).

## Results and discussion

### Characterization of active carbon

**Fig. 1:**
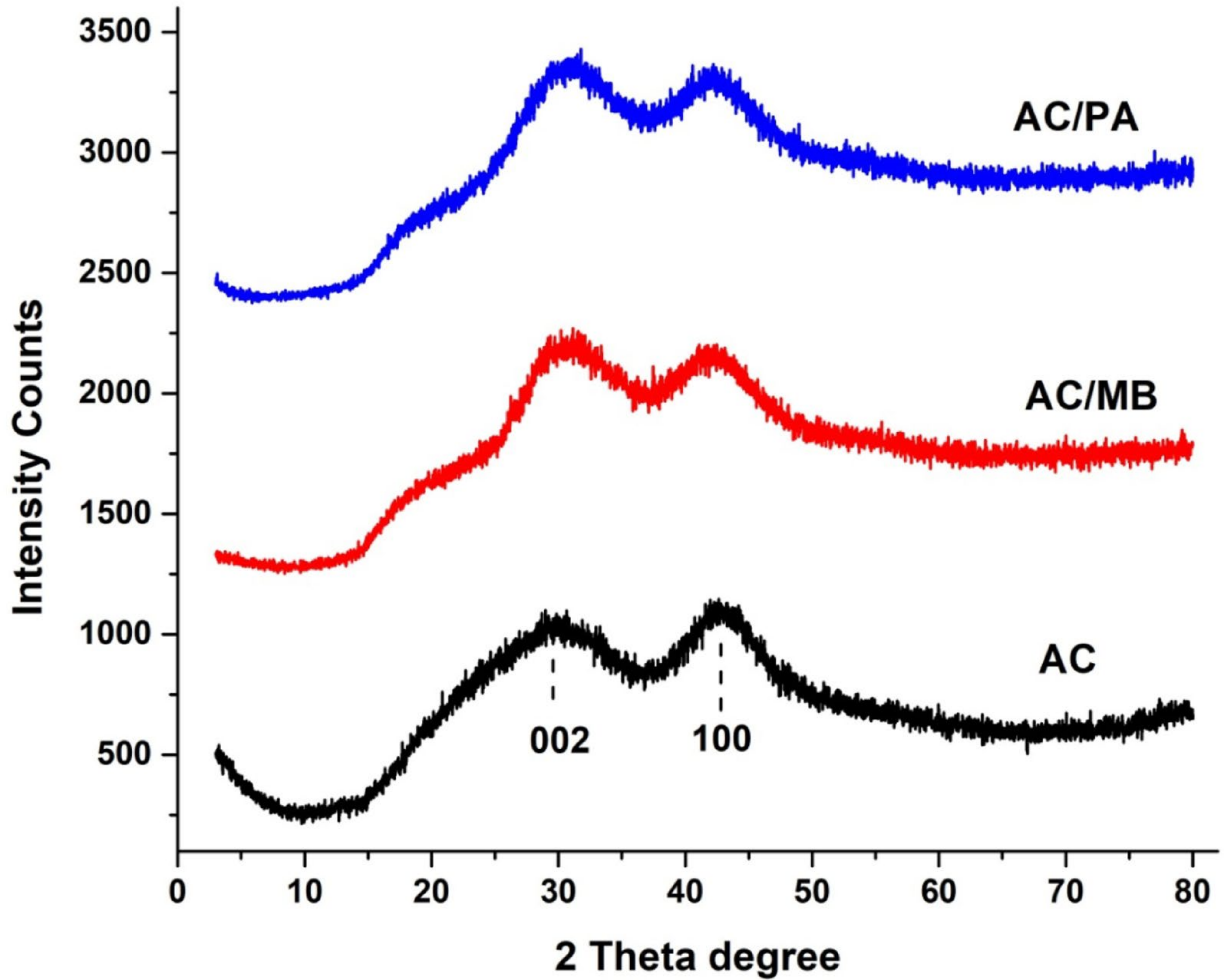
XRD patterns of AC prior to and following MB and PA adsorption

Figure 1 presents the XRD spectra of AC before and after adsorption. The XRD patterns show increased peak intensity in the 10–30° range, suggesting continuous pores that scatter X-ray radiation [26]. Distinct peaks at 2θ = 24.26° and 42.52° correspond to the (002) and (100) crystallographic planes, respectively, matching the JCPDS reference (card no. 00-001-0640). The broad peak shapes indicate low crystallinity and a predominantly amorphous structure [32]. The peaks appear more intense after adsorption, likely due to micropore filling by adsorbate molecules [33]. However, the post-adsorption diffractograms of AC after MB and PA removal show no significant changes, demonstrating that the adsorption process preserves the material’s structural integrity [34].

**Fig. 2:**
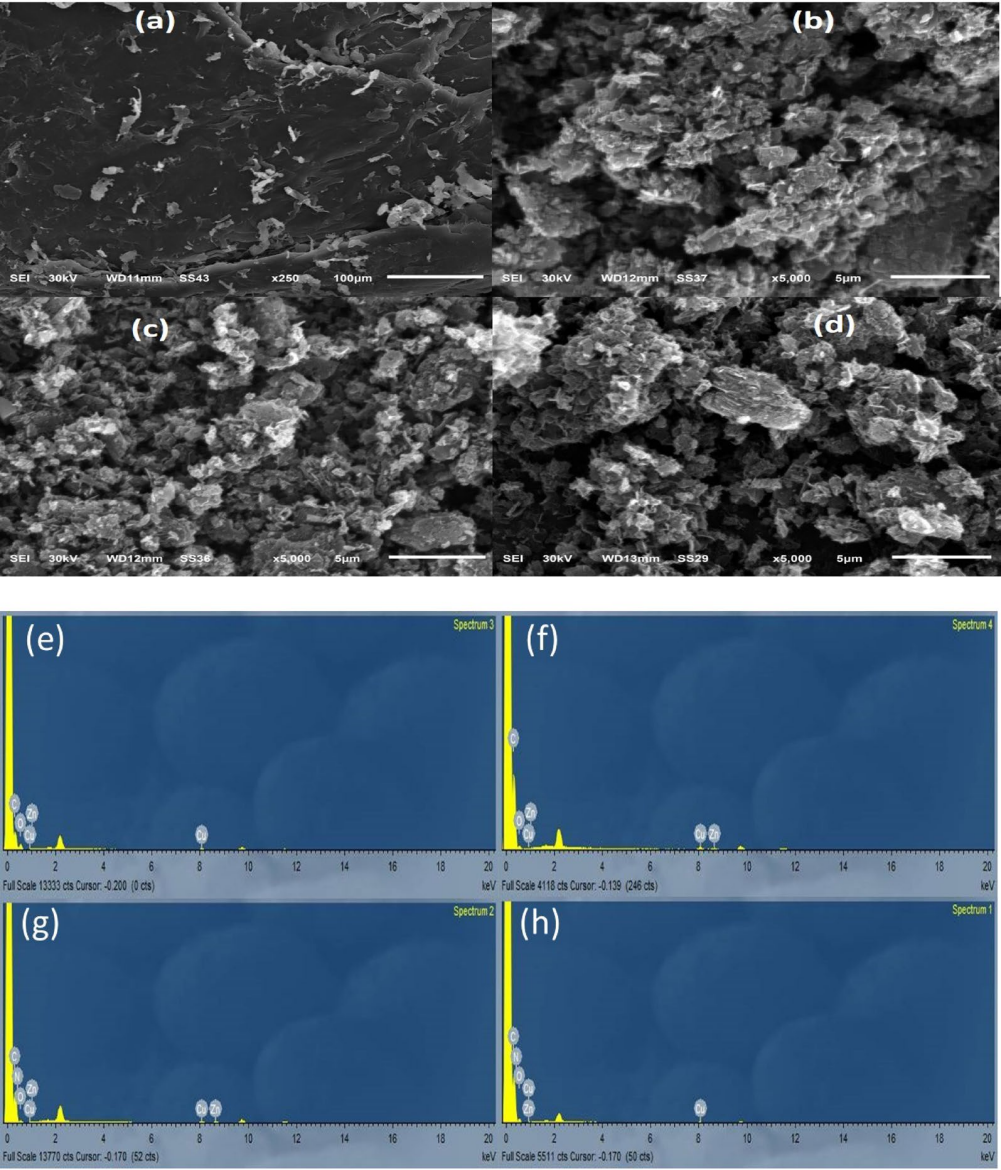
SEM analysis of (**a**) raw PET (**b**) AC (**c**) AC/MB (**d**) AC/PA and EDX analysis of (**e**) raw PET (**f**) AC (**g**) AC/MB (**h**) AC/PA

SEM analysis was performed to compare the surface morphologies of raw PET, AC, and AC after MB and PA adsorption. Figure 2 reveals significant morphological differences before and after analyte uptake. Panels 2(a) and 2(b) demonstrate the surface modification of chemically activated AC compared to raw PET. The activation process substantially enhanced surface roughness and porosity. During carbonization, chemical agent dissolution within the impregnated PET matrix generated abundant pores and voids, resulting in increased surface area and micropore formation. This structural evolution confirms successful activation and improved adsorption capacity. In contrast, raw PET waste maintained a smooth, uniform surface characteristic of polymeric materials [35]. Post-adsorption morphological changes are clearly visible in Fig. 2c and d, where pore blockage and filling evidence effective dye adsorption. The AC surface appeared smoother and less porous due to adsorbate accumulation [36]. Table 2S presents EDX compositional analysis of raw PET, AC, AC/MB, and AC/PA. Activation increased carbon content from 67.16% to 89.12% while reducing oxygen content from 30.76% to 9.06%. Notably, nitrogen peaks in AC/MB (43.34%) and AC/PA (42.37%) spectra confirmed successful dye adsorption by AC.

**Fig. 3:**
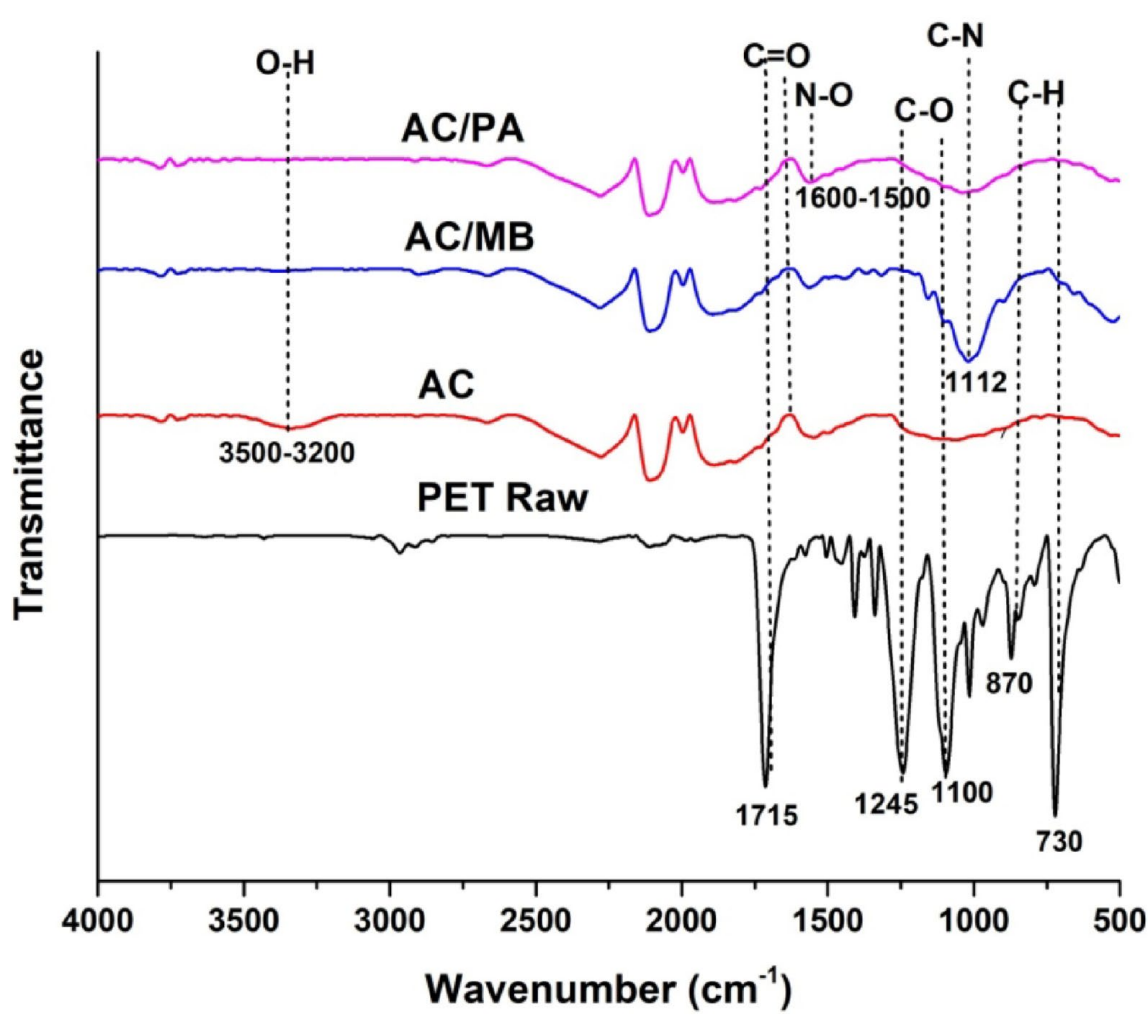
FT-IR of raw PET, AC, and AC after adsorption of MB and PA

Figure 3 presents the FTIR spectra of raw PET and AC. The raw PET spectrum exhibits five characteristic absorption bands: 1715 cm⁻¹ C=O stretching in ester linkages, 1245 cm⁻¹ C–O stretching in aromatic and aliphatic ethers, 1100 cm⁻¹ aromatic C–O stretching, 870 cm⁻¹ out-of-plane aromatic C–H bending, and 730 cm⁻¹ aromatic C–H bending [37].

The AC spectrum before adsorption displays a broad hydroxyl (–OH) stretching vibration at 3500–3200 cm⁻¹. Following adsorption, this peak shifts to ~3400 cm⁻¹ with reduced intensity, suggesting hydroxyl group participation in MB and PA binding. Similarly, carbonyl (C=O) stretching vibrations at 1700–1680 cm⁻¹ exhibit intensity reduction and/or wavenumber shifts, indicating adsorbate interactions [38]. Following MB adsorption, the FTIR spectrum reveals a new sharp peak at ~1338 cm⁻¹ and a distinct band at ~1112 cm⁻¹, the latter corresponding to C–N stretching of the adsorbed dye. Concurrently, the intensities of the O–H (~3400 cm⁻¹) and C=O (~1700 cm⁻¹) bands decrease, while the aromatic C=C band near 1600 cm⁻¹ shifts slightly, indicating surface interactions with MB. For PA adsorption, spectral changes include decreased intensity and shifting in the hydroxyl (~3400 cm⁻¹) and carboxyl (1700–1600 cm⁻¹) regions. Additionally, bands between 1500 and 1600 cm⁻¹, attributed to aromatic C=C stretching, are enhanced or newly formed. The most prominent evidence is the shifting of nitro-group (NO₂) vibrations within the 1550–1350 cm⁻¹ range, confirming PA adsorption. Broadening in the O–H region further suggests interactions involving phenolic hydroxyl groups [39, 40].

The N₂ adsorption-desorption isotherm of AC is classified as Type IV according to IUPAC standards, confirming its mesoporous structure with an average pore radius of 21.4 nm. The isotherm exhibits a distinct Type H3 hysteresis loop, attributed to capillary condensation [41, 42]. At low relative pressures (P/P₀ < 0.2), the adsorbed volume increases gradually, suggesting monolayer adsorption [43]. AC analyzed in Fig. 1S has a BET surface area of 183.7 m² g^− 1^ and a pore volume of 0.9807 cm³ g^− 1^.

**Fig. 4:**
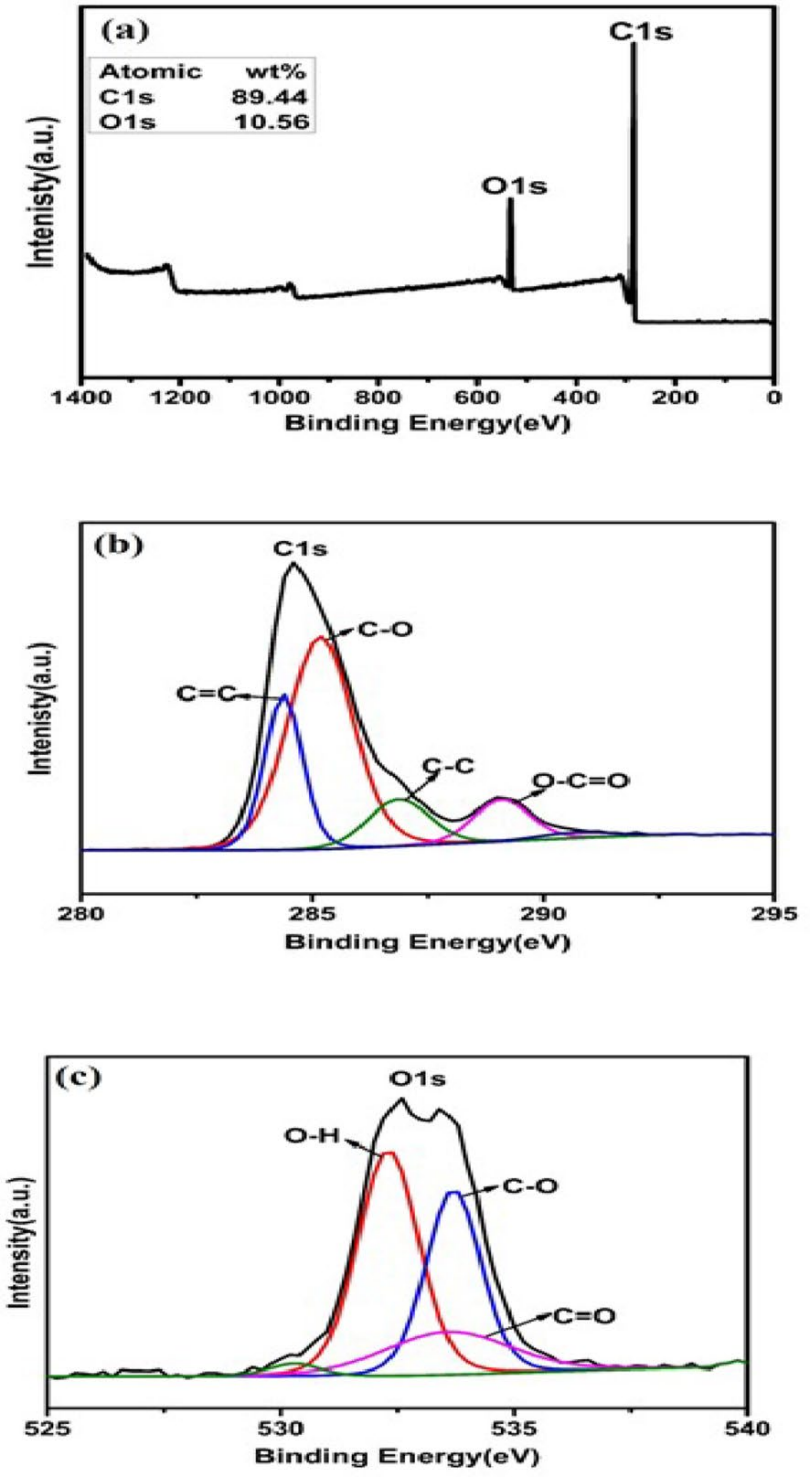
High-resolution XPS spectra of (**a**) AC (**b**) Deconvoluted C 1 s, and (**c**) O 1s

The surface composition and functional groups of AC derived from PET were characterized using XPS. As shown in Fig. 4, the spectra exhibited characteristic peaks for C 1 s and O 1 s, confirming carbon and oxygen as the primary components of AC. Deconvolution of the C 1 s spectra revealed multiple carbon bonding states: C=C (sp²) at 284.4 eV, C–C (sp³) at 286.6 eV, C–O at 285.8 eV, O=–C-OR at 289.9 eV, and a potential carboxyl group contribution at 290.2 eV [44, 45]. The O 1 s spectra displayed peaks corresponding to quinone groups (530.7 eV), carbonyl groups (532.0 eV), and C-O bonds (533.5–533.7.5.7 eV) [46, 47]. The KOH activation process facilitated the formation of these functional groups on the AC surface, thereby modifying its chemical properties.

### Factors influencing the adsorption process

#### Initial pH and point of zero charge

**Fig. 5:**
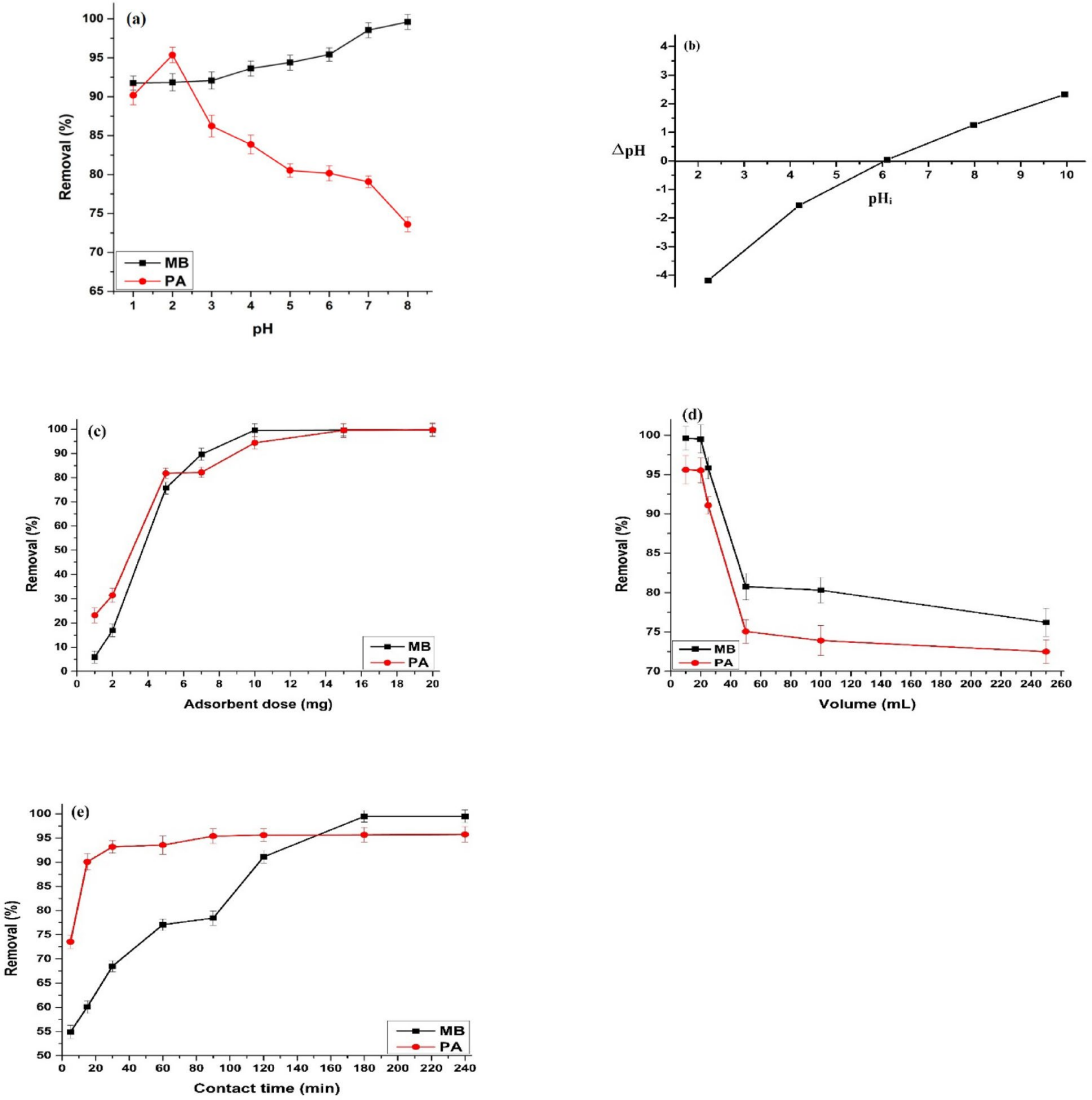
(**a**) Effect of pH on the removal of MB and PA by AC (**b**) pH_PZC_ of AC (**c**) Effect of adsorbent dose (**d**) Effect of volume (**e**) Effect of contact time on the uptake of MB and PA by AC

Figure 5a illustrates the effect of pH on the removal of MB and PA by the prepared AC at an initial concentration of 100 mg L⁻¹, a contact time of 3 h (298 K), and an AC mass of 10 mg. The different adsorption tendencies of MB and PA (Fig. 5a) were elucidated based on the pHzpc of AC (6.1) as shown in Fig. 5b. Above this pH, MB (pK_a_ 3.8) stays positively charged and is strongly attracted to the negatively charged AC surface, but PA (pKa = 0.4) is primarily deprotonated and negatively charged at the working pH, resulting in reduced electrostatic contact and adsorption. Quantitative recovery (95.0–100.0%) of MB at pH 7.0–8.0. Below the pH_pzc_ of AC, the surface charge becomes positive, repelling MB⁺ ions and reducing adsorption efficiency, whereas above the pH_pzc_, the negatively charged surface enhances MB uptake. Additionally, the pKₐ of methylene blue (3.8) marks the transition between its protonated (cationic) and deprotonated forms, with the cationic form dominating at pH < 3.8 [48, 49]. In contrast, PA uptake is quantitative at pH 2.0 due to its higher solubility in acidic conditions. As the pH increases, PA solubility decreases, reducing adsorption efficiency [50]. At lower pH, PA uptake by AC is favored, while at higher pH, the negatively charged or neutral surface functional groups of AC lead to electrostatic repulsion, further diminishing adsorption [51]. Based on these findings, pH 7.0 and 2.0 were selected as optimal for MB and PA removal, respectively.

#### Adsorbent dose

Figure 5c illustrates the effect of AC dosage on the adsorption of MB and PA. The removal efficiencies increased significantly from 5.8% to 99.6% for MB and from 23.1% to 99.7% for PA as the AC mass was increased from 0.001 g to 0.01 g. This enhancement is attributed to the greater availability of active sites and an increased surface area of the sorbent. The optimal sorbent dosage for maximum removal was determined to be 10 mg [31, 52].

#### Impact of sample volume

The removal efficiency of MB and PA by AC is strongly influenced by the solution volume as presented in Fig. 5d. Smaller volumes enhance adsorption by increasing the concentration gradient, thereby strengthening the interaction between the adsorbates and the AC surface. In contrast, larger volumes reduce this gradient, weakening the driving force for adsorption and decreasing efficiency. Experimental results show that a 20 mL sample achieved a preconcentration factor of 10 with a quantitative extraction (> 95% efficiency). However, volumes exceeding 20 mL led to a significant decline in adsorption performance. These findings highlight the need to optimize solution volume for maximum adsorption efficiency [53].

#### Contact time and kinetic studies

The effect of contact time on the adsorption of MB and PA by AC was evaluated over 5–240 min using a 20 mL model solution containing 100 mg L⁻¹ of the analyte at the optimal pH, with a sorbent dose of 0.01 g at ambient temperature. Figure 5e illustrates the uptake of MB and PA as a function of contact time. For MB, the removal efficiency increases rapidly within the first 20 min, followed by a gradual rise until reaching a plateau (99.6%) after 180 min. In contrast, PA exhibited faster adsorption kinetics, achieving over 90% removal within the initial 20 min and reaching equilibrium at 90 min. In both cases, dye removal increased with contact time, showing rapid adsorption during the initial phase, which slowed as equilibrium was approached. This behavior highlights superior efficiency and faster adsorption kinetics of PA compared to MB. This rapid initial adsorption is attributed to strong attractive forces between the adsorbate species and the available active sites on the AC surface. As these sites became occupied, the adsorption rate decreased, eventually stabilizing at equilibrium [54].

The kinetics of MB and PA adsorption onto AC have been thoroughly investigated using pseudo-first-order (PFO: ($\:\mathrm{ln}\left({q}_{e}-{q}_{t}\right)=ln{q}_{e}-{k}_{1}t\:$) and pseudo-second-order (PSO: $\:\frac{t}{{q}_{e}}=\frac{1}{{k}_{2}{q}_{e}^{2}}+\frac{1}{{q}_{e}}\:\:$), and Weber-Morris intra-particle diffusion model ($\:{q}_{t}={K}_{id}.{t}^{0.5}+{C}_{i}$). Figure 2s illustrates the application of these models to the uptake of MB and PA by AC, while the corresponding mathematical formulations provide further details. As shown in Table 3s, the experimental adsorption capacities (q_e_ exp) closely align with the calculated values (q_e_ cal) from the PSO model. Additionally, the correlation coefficient (R^2^) for the PSO model was significantly higher than those for the PFO and intra-particle diffusion models. These findings indicate that the adsorption of MB and PA by AC predominantly follows PSO kinetics, suggesting that the removal process is governed by electron exchange between the adsorbates and the AC surface [39].

#### Adsorption isotherms

Adsorption isotherms are essential for understanding the equilibrium distribution of adsorbed species between the solution and the solid phases, particularly for systems involving MB and PA dyes on AC. Analyzing the adsorption behavior provides valuable insights for optimizing and designing the adsorption process. In this investigation, Langmuir, Freundlich, Temkin, and Dubinin–Radushkevich (D–R) isotherm models were employed to investigate the equilibrium distribution of adsorbed molecules on the solid phase. The Langmuir adsorption model presented in Fig. 3s (a-e) is widely used for studying monolayer adsorption, based on the premise that adsorption occurs at specific, homogeneous sites on the adsorbent surface and described by Eq. 3. Freundlich isotherm model describes the adsorption intensity and capacity of an adsorbate on heterogeneous surfaces; however, it does not provide a precise prediction of the total adsorption capacity [55]. Equation 4 expresses the Freundlich isotherm in linear form. The Freundlich constants, [(mg•g⁻¹)(L•mg⁻¹)]1/nF), are used to determine the adsorption capacity and strength, respectively. Figure 3s (b, f) demonstrates how to calculate (K_F_) and (nF) by plotting (ln qe) against (ln Ce), where the intercept and slope correspond to (K_F_) and (1/nF), respectively. The value of (nF) indicates the degree of nonlinearity between the adsorbent and the solution concentration. When (nF = 1.0), the adsorption is linear, suggesting a chemical adsorption process if (nF < 1), while (nF > 1) indicates that physical adsorption is the dominant mechanism.

The Temkin isotherm model analyzes the indirect interactions between the adsorbent and the adsorbate during the adsorption process. It allows for the determination of key parameters, such as adsorption heat and binding energy, which are uniformly distributed throughout the adsorption process. The isotherm is best represented by the following Eq. 5.

The plot of (q_e_) (mg g⁻¹) against (ln C_e_) shown in Fig. 3s (c) and (g) was used to calculate the Temkin adsorption parameters (b_T_) and (K_T_), which are presented in Table 4S. The (R^2^) value for the Temkin isotherm was lower compared to the Freundlich and Langmuir models, indicating that it was less suitable for describing the adsorption process. Therefore, the Temkin isotherm was primarily useful for comparing the equilibrium behavior with the Freundlich and Langmuir models, rather than for designing the adsorption process. Figure 3s (d)(h) demonstrates the Dubinin–Radushkevich (D–R) isotherm model, applied to describe adsorption on heterogeneous surfaces, was used to elucidate the adsorption mechanism via the mean adsorption energy (E = (2β)⁻¹/²). The calculated E values of 18.6 kJ/mol for MB and 20.7 kJ/mol for PA significantly exceed the threshold for typical chemisorption (8–16 kJ/mol). These elevated energies indicate a adsorption mechanism governed by strong chemical interactions, such as charge transfer or complexation with surface functional groups. Furthermore, the high E values suggest that pore diffusion within the adsorbe.

nt’s complex structure may be a significant rate-controlling step [56]. Based on the regression correlation coefficient (R^2^) values, the Langmuir isotherm model best described the equilibrium data for removing MB and PA onto AC, suggesting a monolayer and homogeneous adsorption process. The prepared AC showed adsorption capacities of 334.4 mg g^−1^ for MB and 271.7 mg g^−1^ for PA, which are superior than that of other sorbents making it an effective sorbent (Table 1).

**Table 1:** Evaluation of adsorption performance of various adsorbents for the removal of MB and PA

Adsorbent	q_max_ (mg g^− 1^)		Ref.
	MB	PA	
Magnetic biochar prepared from common reed	353.4		[57]
Activated carbon derived from sugarcane bagasse	136.5		[58]
Activated carbon prepared from oak seeds	24.0		[55]
Activated carbon from bamboo chip	305.3		[59]
Activated carbon from KOH-activated dragon fruit peels	195.2		[60]
Activated carbon prepared from corn stigmata fibers	330.5		[61]
sugarcane bagasse carbon-Al(OH)3 composite	261.0		[2]
magnetic Pyrus pyrifolia biochar	967.8		[62]
Polymer-functionalized magnetic activated carbon		123.5	[63]
Magnetic activated carbon		72.0	[64]
Silicate MCM-41		327.1	[51]
Zr-Based Metal–Organic Framework		22.5	[65]
Polycation-clay mineral nanocomposites		48.8	[66]
Multi-walled carbon nanotubes/chitosan nanocomposite		157.8	[67]
Activated carbon from KOH-activated PET	334.4	271.7	This study

#### Thermodynamic parameter

Equation 8 has been employed for determining thermodynamic parameters such as change in enthalpy (ΔH^∘^), change in entropy (ΔS^∘^), and change in Gibbs free energy (ΔG^∘^).

9 $$\:\varDelta\:\:\mathrm{G}^\circ\:=-\mathrm{R}\mathrm{T}\:\mathrm{l}\mathrm{n}{\mathrm{K}}_{\mathrm{L}}$$

where (R) represents the general gas constant, (T) is the absolute temperature in Kelvin, and (K_L_) is the equilibrium constant. The van’t Hoff equation, which relates temperature to the equilibrium constant, is expressed as (ln K_L_) versus (1/T), where a linear relationship is expected, as shown in Fig. 4s. The slope and intercept of the van ‘t Hoff plot were used to calculate the values of (∆H˚) and (∆S˚), respectively. Thermodynamic parameters for the adsorption of MB and PA onto AC were calculated and are presented in Table 5s. The negative values of (∆G˚) at all temperatures indicate that the adsorption process is spontaneous and thermodynamically favorable. From the van ‘t Hoff plot, the values of (∆H˚) and (∆S˚) for MB and PA adsorption were −79.2 kJ mol⁻¹ and −215.4 J mol⁻¹ K⁻¹ for MB, and −25.5 kJ mol⁻¹ and −53.8 J mol⁻¹ K⁻¹ for PA. The negative (∆S˚) suggests a decrease in randomness at the solid/solution interface during the adsorption of MB and PA. The negative (∆H˚) values indicate that the adsorption of both dyes onto AC is exothermic. The ΔH˚ value being less than 40 kJ mol-1 suggests that the adsorption of MB and PA onto AC is predominantly physical in nature [68].

#### Desorption and reusability

**Fig. 6:**
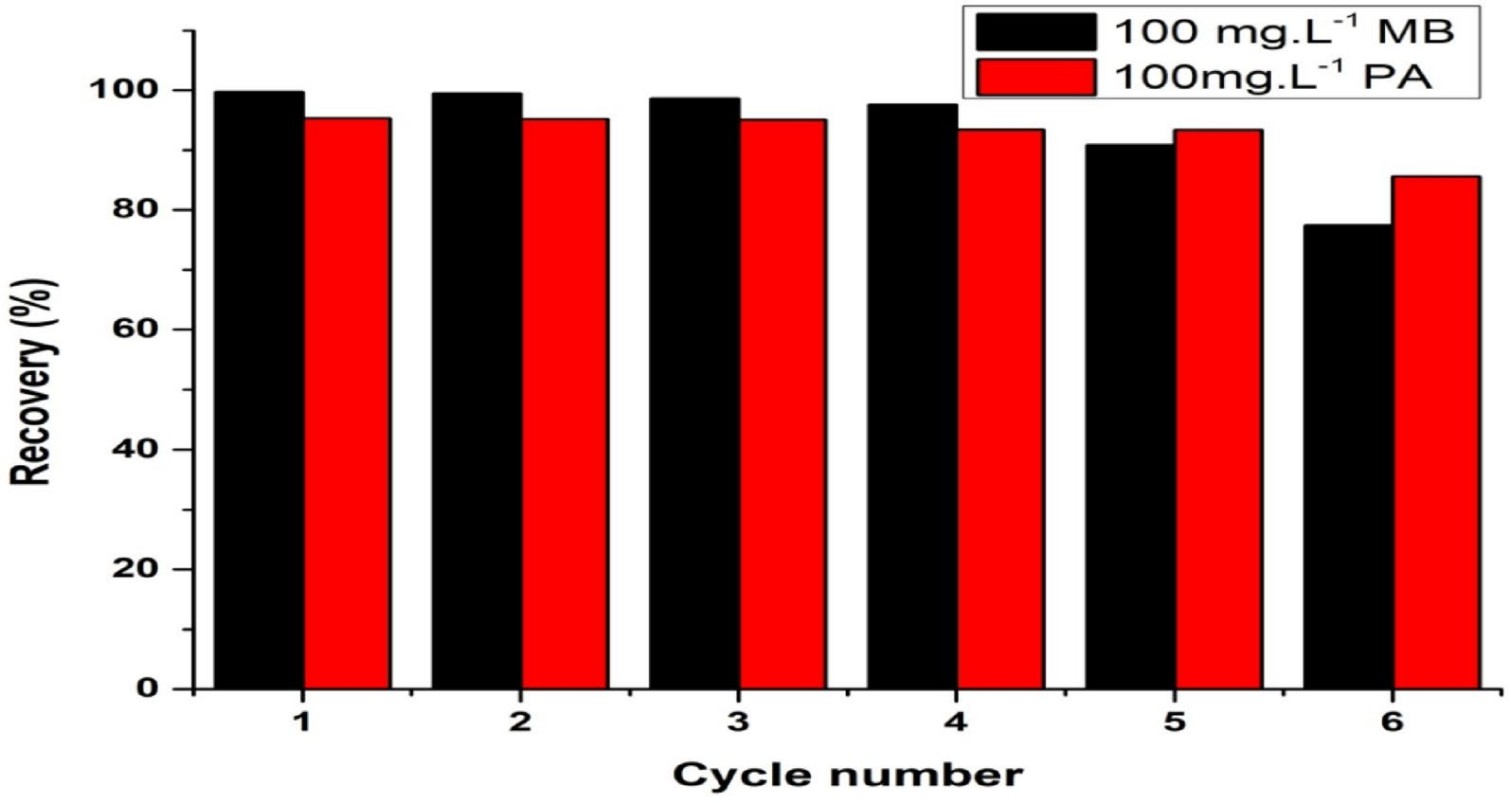
Cycle of regeneration for AC post adsorption of MB and PA

The experiment was performed to determine the ability of AC to retain its adsorption capacity over multiple reuse cycles. It was decided to carry out six consecutive adsorption–desorption cycles (Fig. 6). In each cycle, 10 mg of AC was mixed with 20 mL of a 100 mg L⁻¹ solution of either MB or PA. For desorption, 2.0 mL of 0.5 M H₂SO₄ in the case of MB and 0.5 M NaOH for PA was used. By utilizing several cycles, a decrease in the removal efficiency of PA had taken place from approximately 97.3% to 85.6%; potential reasons could be surface modifications that have taken place or a reduction in active binding sites. In contrast, MB showed good performance during the first five cycles; only a small decline was observed in the last cycle [69, 70]. Regeneration through chemical method enhances AC’s longevity and efficiency, promoting sustainable wastewater treatment practices. These findings confirm AC’s effectiveness as a reusable adsorbent for removing cationic and anionic dyes.

#### Selectivity

The impact of common matrix ions and possible interfering dyes was evaluated to examine the analytical method’s selectivity for MB and PA in ideal circumstances. The maximum concentration at which the extraction recovery did not deviate by more than ±5% was determined to be the tolerance limit. Even in the presence of complex matrices, the method’s high selectivity and low interference from coexisting species ensured precise and dependable extraction of MB and PA, as shown in Table 2.

**Table 2:** Impact of interfering species on the determination of 1.0 Mg L^–1^ of MB and PA

Ions	Tolerance limit (mg L^− 1^) MB PA	
Na^+^, K^+^, Mg^2+^, Ca^2+^, Cl^− 1^, NO_3_^−^, SO_4_^2−^ Ni^2+^, Cd^2+^, Cu^2+^, Zn^2+^ Xylenol Orange Methyl Orange Malachite green, neutral red, Congo red	1000 300 150 300 1	1000 200 4 3 1

#### Analytical figures of merit

The analytical characteristics of the proposed method is summarized in Table 3. Under optimized conditions, the method exhibited linear dynamic ranges for MB (0.15–5.76 mg L⁻¹, A = 0.165 C + 0.032, R^2^ = 0.997) and PA (0.013–1.68 mg L⁻¹, A = 0.876 C + 0.045, R^2^ = 0.998). LOD and LOQ corresponding to signal-to-noise ratios of 3 and 10, respectively were 0.046 and 0.156 mg L⁻¹ for MB and 0.003 and 0.013 mg L⁻¹ for PA. Precision was evaluated through intra-day and inter-day relative standard deviations (%RSDs) at 0.2 mg L⁻¹ and 1.0 mg L⁻¹, with intra-day RSDs of 1.9% for MB and 1.2% for PA at 0.2 mg L⁻¹, and 2.88% for MB and 1.98% for PA at 1.0 mg L⁻¹. Inter-day RSDs were 2.59% for MB and 1.61% for PA at 0.2 mg L⁻¹, 2.93% for MB, and 2.22% for PA at 1.0 mg L⁻¹. Preconcentration factors, determined from calibration curve slopes with and without preconcentration, were 10.1 for MB and 9.6 for PA. The method’s analytical performance is comparable to or exceeds that of previously reported techniques for MB and PA detection and removal (Table 4).

**Table 3:** Analytical properties of the developed method

Parameter	MB	PA
Linear range (mg L^–1^) Calibration regression Eq.(R^2^) Enrichment factor LOD (mg L^− 1^) LOQ (mg L^− 1^)	0.15–5.76 A = 0.165 C + 0.032 0.997 10.09 0.046 0.156	0.013–1.68 A = 0.876 C + 0.045 0.998 9.56 0.003 0.013
Intra-day RSD (%)		
0.2 (mg L^–1^) 1.0 (mg L^–1^)	1.92 2.88	1.28 1.98
Inter-day RSD (%)		
0.2 (mg L^–1^) 1.0 (mg L^–1^)	2.59 2.93	1.61 2.22

**Table 4:** Comparative analysis of analytical characteristics across different extraction techniques for MB and PA

Extraction procedure	Analyte	Linearity	LOD	PF	RSD%	Ref
Dispersive solid phase microextraction based on magnesium oxide nanoparticles	MB	5–2000 µg L^–1^	1.66 µg L^–1^	44.5	between 2.9% and 3.1%	[71]
Ultrasonic-assisted magnetic solid phase extraction	MB	40–450 µg L^–1^	11 µg L^–1^	40	2.3%	[72]
Combined dispersive solid phase and cloud point extraction using Cu(OH)_2_ nanoflakes	MB	2.0–350.0.0.0 µg L^–1^	0.65 µg L^–1^	120	1.05% and 1.65%	[73]
Liquid–liquid extraction based on dual-valve sequential injection system.	PA	7.65–115 mg L^− 1^	1.06 mg L^− 1^	-	Between 0.8% and 1.2%	[74]
Preconcentration based on magnetic nanoparticles coated with cetyltrimethylammonium bromide	PA	0.02–1.00 mg L^− 1^	0.007 mg L^− 1^	50	3.98% and 1.97%	[75]
Preconcentration based on activated carbon	MB	0.15–5.76 mg L^− 1^	0.046 mg L^− 1^	10.09	Between 1.92% and 2.93%	Our study
Preconcentration based on activated carbon	PA	0.013–1.68 mg L^− 1^	0.003 mg L^− 1^	9.56	Between 1.28% and 2.22%	Our study

#### Method validation to water samples

The feasibility and applicability of the developed method were assessed by spectrophotometric analysis of various water samples, including treated sewage water, tap water, and river water. The accuracy of the process was assessed using the analysis of spiked samples. Table 5 presents recovery values for spiked samples and demonstrates method validation. As shown, changes in the sample matrix did not significantly influence the extraction efficiency of MB and PA. With relative recoveries ranging from 95.4% to 104.9%, the method demonstrated high accuracy, efficiency, and reliability across different water matrices.

**Table 5:** Method validation in various water samples

Dye	Sample	Added (mg L^− 1^)	Found (mg L^− 1^)	Recovery (%)	RSD (%)
MB	Treated Sewage Water	0 1 2 3	Not detected 0.95 ± 0.025 1.96 ± 0.021 2.96 ± 0.015	- 95.38 98.41 98.81	- 2.63 1.07 0.51
	River Water	0 1 2 3	Not detected 0.96 ± 0.015 1.99 ± 0.012 3.04 ± 0.021	- 96.39 99.82 101.36	- 1.65 0.60 0.68
	Tap Water	0 1 2 3	Not detected 0.96 ± 0.012 2.05 ± 0.019 3.14 ± 0.009	- 96.79 102.94 104.85	- 1.29 0.94 0.29
PA	Treated Sewage Water	0 0.5 1	Not detected 0.47 ± 0.013 0.96 ± 0.016	- 95.91 96.08	- 2.85 1.72
	River Water	0 0.5 1	Not detected 0.48 ± 0.012 1.02 ± 0.010	- 97.74 102.28	- 2.49 1.06
	Tap Water	0 0.5 1	Not detected 0.51 ± 0.013 1.03 ± 0.015	- 102.68 103.57	- 2.59 1.21

Results are expressed as mean ± standard deviation based on three independent measurements

### Proposed mechanism of adsorption

**Fig. 7:**
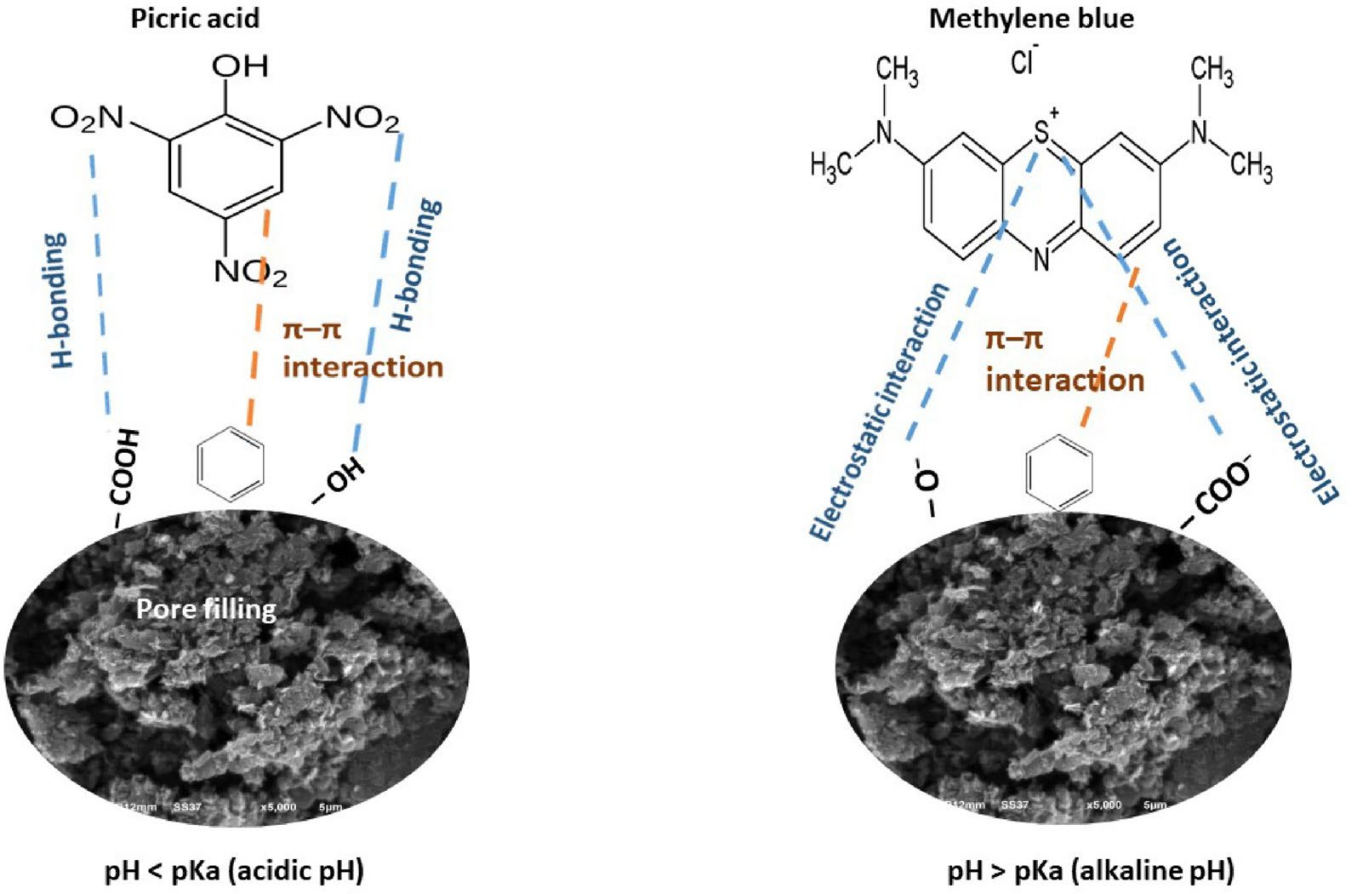
Proposed adsorption mechanism of MB and PA onto AC

MB and PA were adsorbed onto AC using a combination of van der Waals interaction, π-π stacking, hydrogen bonding, pore filling, and electrostatic forces as illustrated in Fig. 7. One important aspect influencing this process is the AC’s point of zero charge (PZC), which is 6.1. Thus, it determines whether the surface of the AC is positively or negatively charged. MB is a positively charged dye that adsorbs better at pH values over 6.1. At these pH levels, the cationic MB molecules are electrostatically attracted to the AC surface because of its negative charge. The π-π stacking between the aromatic rings of MB and the carbon’s electron system, in addition to this attraction, and the capacity of AC’s pores to physically trap MB molecules [76, 77]. However, PA, which is negatively charged, is better absorbed when the pH is below 6.1. In these conditions, PA molecules are more readily attracted to the AC surface electrostatically due to its positive charge. The hydrogen bonding between the oxygen-containing groups on the surface of AC and the functional groups of PA, like -OH and -NO_2_, is another crucial element in the adsorption process [78]. Hydroxyl groups, which can function as hydrogen bond donors or acceptors, were detected on the AC surface by FTIR spectroscopic investigation. Shifts in the FTIR spectrum following the adsorption of MB and PA verified that hydrogen bonding was involved in the process. The adsorption process is exothermic, according to thermodynamic studies, with enthalpy changes (ΔH) of −25.5 kJ.mol^− 1^ for MB and −5.71 kJ.mol^− 1^ for PA. van der Waals forces, among other mechanisms, are indicated by these values.

## Conclusion

This study demonstrates an efficient and sustainable method for converting PET waste into high-performance AC for the removal and subsequent preconcentration of MB and PA. The adsorbent showed high adsorption capacities (334.4 mg g⁻¹ for MB and 271.7 mg g⁻¹ for PA), and the adsorption process followed the Langmuir isotherm and pseudo-second-order kinetics. Thermodynamic results confirmed a spontaneous and exothermic process. The method exhibited excellent analytical performance, including a wide linear range, low limits of detection and quantification (LOD/LOQ), high enrichment factors (10.09 for MB and 9.56 for PA), and low intra- and inter-day RSDs (< 3%). High selectivity was achieved, with tolerance limits up to 1000 mg L⁻¹ for common ions and minimal interference from structurally related dyes. Application to environmental water samples (treated sewage, river, and tap water) yielded recoveries of 95.38–104.85% for MB and 95.91–103.57% for PA, confirming the method’s accuracy, precision, and practical applicability. Overall, this work provides a cost-effective and environmentally friendly strategy for both dye removal and trace-level monitoring in environmental waters.

## Supplementary Information

Supplementary Material 1

## Data Availability

The authors declare that the data supporting the findings of this study are available within the paper.
