# Supplementary material for: The utilization of activated carbon derived from polyethylene terephthalate bottle waste, as a sustainable source for removal and recovery of methylene blue and picric acid from aqueous solutions
Source: BMC Chem. 2026 Feb 28;20(1):73. doi: 10.1186/s13065-026-01728-x (PMC13059600; doi:10.1186/s13065-026-01728-x)
Supplement: Supplementary file 1 — Supplementary Material 1 [file 13065_2026_1728_MOESM1_ESM.docx]

**Supplementary Information**

**The utilization of activated carbon derived from polyethylene terephthalate bottle waste, as a sustainable source for removal and recovery of methylene blue and picric acid from aqueous solutions**

Asmaa Halima (0009-0007-8372-5419) ^a^, Magdi E. Khalifa ^a^, Nasser Mohammed Hosny (0000-0003-1035-1480) ^b^, Wael I. Mortada (0000-0001-7797-0302) ^c,*^

^a^ Department of Chemistry, Faculty of Science, Mansoura University, Mansoura 35516, Egypt

^b^ Chemistry Department, Faculty of Science, Port Said University, P.O. Box 4252, Port-Said, Egypt

^c^ Urology and Nephrology Center, Mansoura University, Mansoura 35516, Egypt

* Corresponding author:

Dr. Wael I. Mortada,

Urology and Nephrology Center, Mansoura University, Mansoura 35516, Egypt,

E-mail: [w.mortada@mans.edu.eg](mailto:w.mortada@mans.edu.eg)

**Fig. 1S** N2 adsorption/desorption isotherm of AC at 77 K including BJH pore size distribution curve

**Fig. 2S**	Adsorption kinetics include the pseudo-first-order model for (a) MB and (d) PA, the pseudo-second-order model for (b) MB and (e) PA, and the intra-particle diffusion model for the adsorption of (c) MB and (f) PA by AC

**Fig. 3S** Adsorption isotherms include the Langmuir model of (a) MB dye; (e) PA dye, the Freundlich model of (b) MB dye; (f) PA dye, the Temkin model for the adsorption of (c) MB dye; (g) PA dye, and Dubinin-Radushkevich (D-R) Isotherm of (d) MB dye; (h) PA dye by AC

**Fig. 4S** Van't Hoff plots illustrating the adsorption of (a) MB dye and (b) PA onto AC

**Table 1s** Characteristic analysis of real water samples

| Items | Nile river water | Tap water | Sewage wastewater |
| --- | --- | --- | --- |
| pH | 6.8 | 7.2 | 8.5 |
| Turbidity (NTU) | 4.46 | 5.6 | 11.4 |
| TDS (mg L^-1^) | 227 | 356 | 1227 |
| Alkalinity CaCO_3_ (mg L^-1^) | 116 | 219 | 75.2 |
| Total hardness (mg L^-1^) | 256 | 231 | 368 |
| NH_3_ (mg L^-1^) | 0.67 | 0.24 | 36 |
| NO_3_ (mg L^-1^) | 2.16 | 2.61 | 0.212 |
| NO_2_ (mg L^-1^) | 0.039 | 0.016 | 0.04 |
| Chloride (mg L^-1^) | 37 | 190 | 54.9 |
| Sulfate (mg L^-1^) | 32 | 37 | 53 |
| Phosphate (mg L^-1^) | 1.86 | 0.04 | 4.72 |

**Table 2S** Elemental analysis (mass %) using EDX for raw PET, AC, and AC after adsorption of MB and PA

| Element | Raw PET | Active Carbon | AC/MB | AC/PA |
| --- | --- | --- | --- | --- |
| C | 67.16 | 89.12 | 41.84 | 43.07 |
| O | 30.76 | 9.06 | 13.88 | 14.14 |
| N | - | - | 43.34 | 42.37 |

**Table 3S** The adsorption kinetics parameters for the uptake of MB and PA by AC

| Kinetic Models | Parameters | MB | PA |
| --- | --- | --- | --- |
| Pseudo-First-Order | q_e_, cal (mg g^-1^)  K_1_ (min^-1^)  R^2^ | 170.02  0.26 ×10^-3^  0.866 | 13.8  0.134 ×10^-3^  0.689 |
| Pseudo-Second Order | q_e_, cal (mg g^-1^)  k_2_ (g. mg^-1^. min^-1^)  R^2^ | 208.7  1.65×10^-6^  0.989 | 192.6  1.705×10^-7^  0.999 |
| Intra-particle diffusion | C_i_ (mg g^−1^)  K_id_ (mg g^-1^min^0.05^)  R^2^ | 95.014  7.2  0.964 | 163.004  2.3  0.439 |
| q_e_,exp |  | 199.2 | 192.4 |

**Table 4S** Adsorption isotherm parameters for MB and PA on AC

| PA | MB | Parameters | Adsorption Isotherm |
| --- | --- | --- | --- |
| 271.7  2.3  237.9  0.987 | 334.4  2.5  257.5  0.997 | q_max_ (mg g^−1^)  K_L_ (L. mg^-1^)  R_L_  R^2^ | **Langmuir** |
| 115.9  0.208  0.845 | 132.2  0.197  0.791 | K_F_ (L. mg^-1^)  1/n  R^2^ | **Freundlich** |
| 32.02  91.4  0.890 | 32.8  155.8  0.900 | B_T_  K_T_  R^2^ | **Temkin** |
| 20.7  1.1E-09  0.0027  0.645 | 18.6  1.4E-09 0.0018  0.837 | E(kJ mol^-1^) β (mol².J^-^²)  q_m_ (mg.g^-1^)  R^2^ | **Dubinin-Radushkevich (D-R) Isotherm** |

**Table 5S** Thermodynamic parameters associated with MB and PA removal on AC

|  | Temperature(K) | Parameters | MB | PA |
| --- | --- | --- | --- | --- |
| Thermodynamic | 298  308  318  328 | ΔH˚ (kJ mol^-1^) | -79.2 | -25.5 |
|  |  | ∆S˚(J mol^-1^ K^-1^) | -215.4 | -53.8 |
|  |  | ΔG˚ ( kJ mol^-1^ ) | -16.4  -10.9  -9.9  -9.8 | -9.8  -9.6  -7.4  -8.8 |
